# Early detection of Niemann-pick disease type C with cataplexy and orexin levels: continuous observation with and without Miglustat

**DOI:** 10.1186/s13023-020-01531-4

**Published:** 2020-09-29

**Authors:** A. Imanishi, T. Kawazoe, Y. Hamada, T. Kumagai, K. Tsutsui, N. Sakai, K. Eto, A. Noguchi, T. Shimizu, T. Takahashi, G. Han, K. Mishima, T. Kanbayashi, H. Kondo

**Affiliations:** 1 Department of Psychiatry, Akita University Graduate School of Medicine, Akita, Japan; 2 Department of Neurology, National Center of Neurology and Psychiatry, Tokyo, Japan; 3 Department of Pediatrics, Toyonaka Municipal Hospital, Toyonaka, Japan; 4 National Center for Child Health and Development, Tokyo, Japan; 5 Division of Health Sciences, Osaka University Graduate School of Medicine, Osaka, Japan; 6 Department of Pediatrics, Tokyo Women’s Medical University, Tokyo, Japan; 7 Department of Pediatrics, Akita University Graduate School of Medicine, Akita, Japan; 8 Akita Mental Health and Welfare Center, Akita, Japan; 9 International Institute for Integrative Sleep Medicine (IIIS), University of Tsukuba, Tsukuba, 305-8575 Japan

**Keywords:** Niemann-pick type C (NPC), Narcolepsy, Cataplexy, Orexin (hypocretin), Miglustat

## Abstract

**Study objectives:**

Niemann-Pick type C (NPC) is an autosomal recessive and congenital neurological disorder characterized by the accumulation of cholesterol and glycosphingolipids. Symptoms include hepatosplenomegaly, vertical supranuclear saccadic palsy, ataxia, dystonia, and dementia. Some cases frequently display narcolepsy-like symptoms, including cataplexy which was reported in 26% of all NPC patients and was more often recorded among late-infantile onset (50%) and juvenile onset (38%) patients. In this current study, we examined CSF orexin levels in the 10 patients of NPC with and without cataplexy, which supports previous findings.

**Methods:**

Ten patients with NPC were included in the study (5 males and 5 females). NPC diagnosis was biochemically confirmed in all 10 patients, from which 8 patients with NPC1 gene were identified. We compared CSF orexin levels among NPC, narcoleptic and idiopathic hypersomnia patients.

**Results:**

Six NPC patients with cataplexy had low or intermediate orexin levels. In 4 cases without cataplexy, their orexin levels were normal. In 5 cases with Miglustat treatment, their symptoms stabilized or improved. For cases without Miglustat treatment, their conditions worsened generally. The CSF orexin levels of NPC patients were significantly higher than those of patients with narcolepsy-cataplexy and lower than those of patients with idiopathic hypersomnia, which was considered as the control group with normal CSF orexin levels.

**Discussion:**

Our study indicates that orexin level measurements can be an early alert of potential NPC. Low or intermediate orexin levels could further decrease due to reduction in the neuronal function in the orexin system, accelerating the patients’ NPC pathophysiology. However with Miglustat treatment, the orexin levels stabilized or improved, along with other general symptoms. Although the circuitry is unclear, this supports that orexin system is indeed involved in narcolepsy-cataplexy in NPC patients.

**Conclusion:**

The NPC patients with cataplexy had low or intermediate orexin levels. In the cases without cataplexy, their orexin levels were normal. Our study suggests that orexin measurements can serve as an early alert for potential NPC; furthermore, they could be a marker of therapy monitoring during a treatment.

## Introduction

Niemann-Pick type C (NPC) is an autosomal recessive and congenital neurological disorder characterized by the accumulation of cholesterol and glycosphingolipids in the peripheral tissues and in the brain [1–5]. NPC is associated with mutations in NPC1 and NPC2 genes with a minimal incidence calculated as 1:150,000 live births. Symptoms include hepatosplenomegaly, vertical supranuclear saccadic palsy (VSSP), ataxia, dystonia, and dementia. Some cases frequently display narcolepsy-like symptoms, including cataplexy (about 26%) [6]. Cataplexy is often triggered by typical emotions (laughing, enjoying, joking and anger). This disease is categorized from the age at onset of neurological symptoms, peri/pronatal, early-infantile, late-infantile, juvenile and adolescent/adult-onset period [6]. Not until recently, miglustat, an effective pharmacological treatment, has been used from 2009 in EU and 2012 in Japan. However, early diagnosis is paramount for starting this treatment [6].

Cataplexy is defined as more than one episode of generally brief (< 2 min), usually bilaterally symmetrical sudden loss of muscle tone with retained consciousness, according to the third edition of the International Classification of Sleep Disorders (ICSD-3) [7]. The episodes are precipitated by strong emotions, usually positive, with almost all patients reporting some episodes precipitated by emotions associated with laughter. The finding of transient reversible loss of deep tendon reflexes during an attack, if observed, is a strong diagnostic indication. In children, cataplexy may present close to disease onset as facial (or generalized) hypotonia with droopy eyelids, mouth opening, and protruded tongue, or gait unsteadiness, which clearly are not related to emotion. Facial and masticatory movements may occur due to muscle weakness. In children, anticipation of a reward is a common precipitant. It is important to use child-appropriate contexts and language when trying to elicit a history of cataplexy.

Cataplexy is a noticeable symptom that suspects to be narcolepsy-cataplexy (=narcolepsy type1, NT1). Being a rare case, diagnosing the patients at an early stage has proven to be difficult. Had the physicians prior knowledge of cataplexy and suspected the disorder to be narcolepsy-cataplexy and relation to NPC in pre-pubertal children, patients would be diagnosed promptly. Cataplexy is reported in 26% of all NPC patients and is more often recorded among late-infantile onset (50%) and juvenile onset (38%) patients, compared with early-infantile onset (18%) and adolescent/adult-onset (5%) patients [6].

In recent NPC suspicion index [8], cataplexy is a novel symptom and continues to progress to the next stage. However, because of its rarity, diagnosis of cataplexy at an early stage with the NPC suspicion index has not been used in due time. Therefore, in an effort to share the knowledge and broaden the perspective, we introduce cataplexy: its pathophysiology of orexin system and manifestation of NPC. In this current study, we examined CSF orexin levels in the 10 patients of NPC with and without cataplexy, which reports a novel and useful information which indicates that orexin measurements can serve an early alert for potential NPC .

## Method

### Patients and procedures

Ten patients with NPC were included in the study (5 males and 5 females). Female average age was 18.6 years (2 y-32 y) and male average age was 11 years (2 y-26 y). We checked for clinical symptoms: brain MRI, HLA and measured orexin levels. In this study, previous 1–5 cases were untreated by miglustat, and recent 6–10 cases were treated by this drug. NPC diagnosis was biochemically confirmed in all 10 patients and among those, 8 patients with NPC1 were identified. The clinical types included in this study were early-infantile, late-infantile, juvenile and adolescent/adult-onset period.

#### Representative case report

(Case 6) A 6 year-old girl was pointed out with development retardation when she was 7 months. She was suspected of having NPC due to the presence of splenomegaly and cataplexy at 3 years. She had no family history of NPC. She was diagnosed with NPC by Niemann-Pick cells in a bone marrow examination and filipin-cholesterol staining in cultured fibroblasts. Her orexin level of CSF was 183 pg/ml, intermediate level. She started Miglustat treatment from June, 2012. One year after treatment, the frequency of cataplexy was decreased, dysphagia, language ability and splenomegaly got better. Orexin level of CSF was increased to 351 pg/ml, normal level, in the duration of this 1 year.

For age matched control comparison, we identified narcolepsy and idiopathic hypersomnia (IHS) patients with previously measured CSF orexin levels. Narcolepsy- cataplexy patients’ ages ranged from 9 to 45 years, while IHS patients’ were from 13 to 49 years. Patients without complete records were excluded. A total of 26 patients with narcolepsy-cataplexy (NT1) and 30 patients with IHS were also enrolled and they were diagnosed according to ICSD-3 [7].

All patients with narcolepsy exhibited EDS for a minimum of 3 months, as well as either a low level of CSF orexin (110 pg/ml or lower) or cataplexy, mean sleep latency of 8 min, or two or more sleep-onset REM periods on a multiple sleep latency test (MSLT). Patients with IHS exhibited EDS for a minimum of 3 months, normal orexin levels, no cataplexy and mean sleep latency of 8 min in MSLT.

Because lumbar puncture is an invasive examination to get CSF for orexin measurements, it was very difficult to gather healthy people for this particular research. Since IHS patients showed normal orexin levels, they were good candidates to serve as control.

All patients were of Japanese ethnicity. Written informed consent was obtained from all patients and/or their guardians. This study was approved by the ethics committee of the Akita University School of Medicine. Some cases [1, 3–5, 7, 9] were previously reported and referenced on Table 1 [10–12].

**Table 1:**
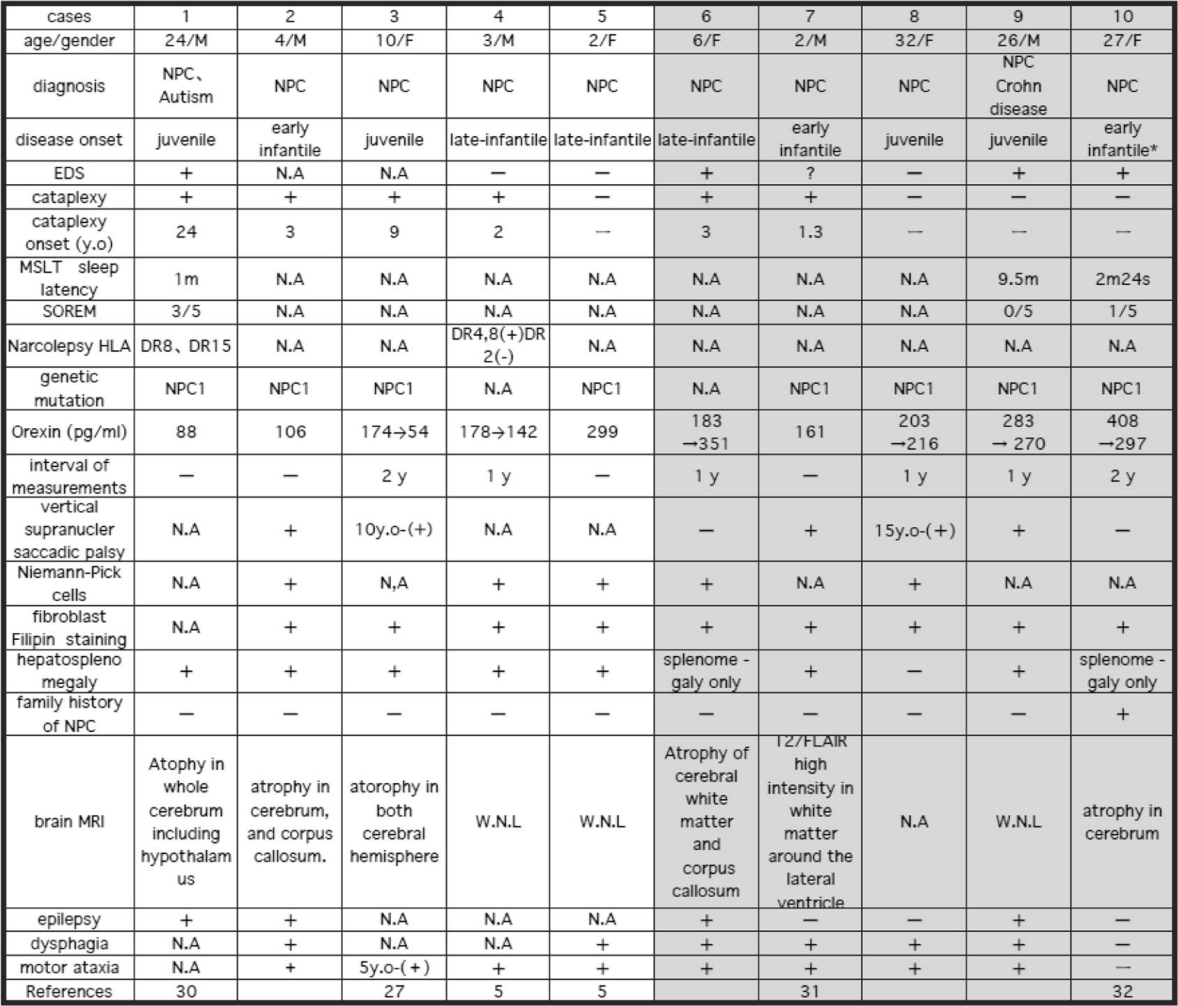
Case profiles: Patients Diagnose, Clinical Symptoms and Orexin Measurements

*This patient was considered to be diagnosed with NPC at early infantile, however compared with other parents, she survived well without neurological symptoms until her late twenties

Cases 6–10 are treated with miglustat, indicated by the gray background

### Measurement of CSF Orexin-a levels

CSF by lumbar puncture was collected between 10:00 and 16:00 in those who consented to the procedure. CSF samples were frozen immediately and stored at − 80 °C for analysis between 2 weeks to12 months. CSF orexin-A levels were measured using a commercially available 125I radioimmunoassay kit (Phoenix Pharmaceuticals, Belmont, CA) as previously described [9, 13]. The orexin limit of detection was arbitrarily set to 40 pg/ml. It has been reported that there was no significant differences in mean CSF orexin levels owing to gender or age [14]. CSF orexin levels were defined as low (110 pg/ml), intermediate (> 110 to 200 pg/ml), or normal (> 200 pg/ml) [15]. Daytime sleepiness was assessed using the Epworth sleepiness scale (ESS) [16]. ESS data were available from 2 NPC patients.

### Statistical analysis

Data were reported as mean ± standard deviation or median (25–75%). Since CSF orexin levels did not show homogeneity of variances, the differences among patients with NPC, narcolepsy-cataplexy (NT1) and IHS were analyzed using Kruskal-Wallis test. When the *p*-value in Kruskal-Wallis test was statistically significant, Mann–Whitney *U* test was performed to compare groups. Mann–Whitney *U* test was followed by Bonferroni post-hoc test. All analyses, along with the calculation of two-sided *p*-values, were performed using IBM SPSS statistics version 24, and the significance level was set at *p* 0.05.

## Result

**Fig. 1:**
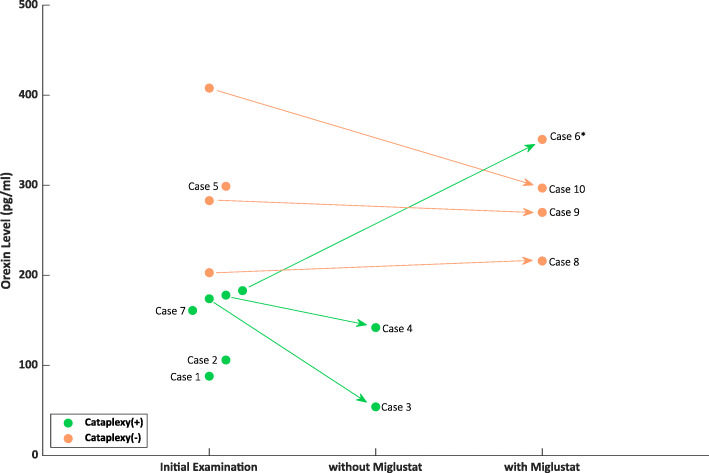
Transitions of CSF orexin levels in initial and follow up examinations of NPC patients. 6 cases with cataplexy exhibit low (< 110 pg/ml) or intermediate (110-200 pg/ml) orexin levels, while 4 cases without cataplexy exhibit normal orexin levels. In two cases with cataplexy without miglustat treatment (case 3, 4), orexin levels at the onset were intermediate, and became lower in the later period (174→54 pg/ml, 178→142 pg/ml). Among 5 cases with miglustat treatment, cataplexy of case 6 disappeared and orexin level increased (183→351 pg/ml). Three other cases without cataplexy remained normal orexin levels during miglustat treatment (203→216 pg/ml, 283→270 pg/ml, 408→297 pg/ml). *As for case 6, cataplexy disappeared with the course of miglustat treatment; thus it is indicated as Cataplexy - (orange)

A summary of data from the 10 NPC patients is shown in Table 1. Case 1–5 were not treated by Miglustat and case 6–10 were treated (Fig. 1). Female average age was 18.6 years and male average age was 11 years. Their age was described at the lumber punctures.

Six out of 10 cases had cataplexy (Table 1, Fig. 1). Their onsets of diseases were early-infantile to juvenile. These 6 cases who had cataplexy exhibited low (< 110 pg/ml) or intermediate (110-200 pg/ml) orexin levels. In two cases with cataplexy (case 3, 4), orexin levels at the onset were intermediate, and became lower in the later period (174→54 pg/ml, 178→142 pg/ml) without miglustat treatment. Miglustat either improved patients’ symptoms or prevented them from worsening for 5 cases. In case 6 cataplexy disappeared and orexin level increased (183→351 pg/ml). Three other cases without cataplexy remained as normal orexin levels (203→216 pg/ml, 283→270 pg/ml, 408→297 pg/ml).

**Fig. 2:**
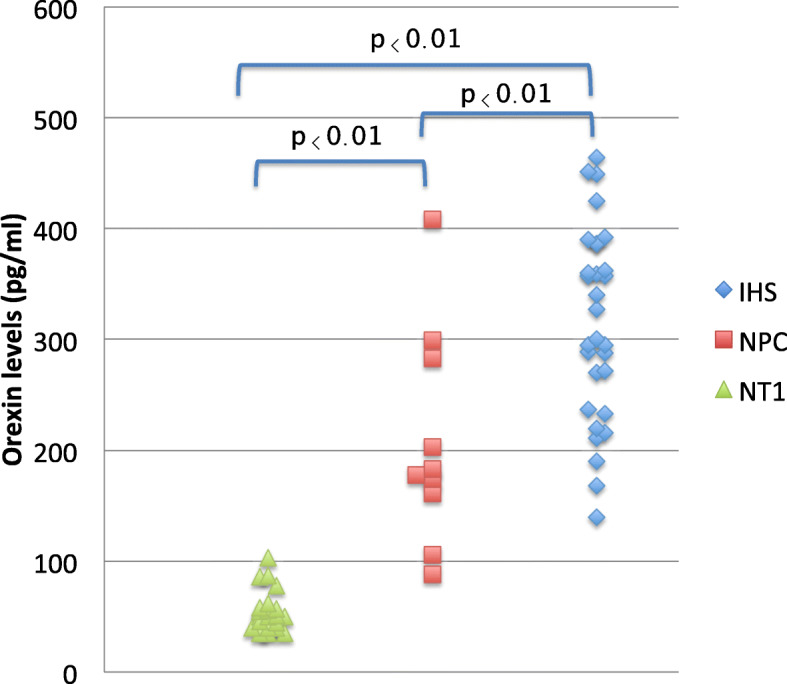
Orexin levels in narcolepsy type1 (NT1), NPC and idiopathic hypersomnia (IHS). The CSF orexin levels of NPC patients were significantly higher than those of patients with narcolepsy type 1 (*p* < 0.01) and lower than those of patients with idiopathic hypersomnia (*p* < 0.01), considered as the control group with normal CSF orexin levels

When comparing among patients with narcolepsy-cataplexy (NT1), NPC and IHS, the median of the CSF orexin levels were 42.5 pg/mL (25–75%: 40, 58.8 pg/ml), 205 pg/mL (139, 239 pg/ml), 292 pg/mL (242.3, 361.5 pg/ml) respectively (Fig. 2). The CSF orexin levels of NPC patients were significantly higher than those of patients with narcolepsy-cataplexy (NT1)(*p* < 0.01) and lower than those of patients with IHS (*p* < 0.01), which was considered as the control group with normal CSF orexin levels.

Compared to cataplexy, EDS was shown in 3 cases. Only case 1 had both cataplexy and EDS. VSSP, one of salient neurological symptoms of NPC, was found in 5 cases. Three cases had both VSSP and cataplexy. NPC diagnosis was biochemically confirmed in all 10 patients, from which 8 patients with NPC1 gene were identified. Only one case had a family history of NPC. Brain atrophies were found in 4 cases and one case showed T2 and FLAIR high intensity around the lateral ventricles by MRI. Hepato-splenomegaly and splenomegaly were found in 9 cases. As for other symptoms, epilepsy in 4 cases, dysphagia in 6 cases and motor ataxia in 8 cases, were found.

## Discussion

We presented 10 Japanese cases of NPC with and without cataplexy. All cases were measured with orexin levels and 6 cases with cataplexy had low or intermediate orexin levels. Some of these 10 cases were early diagnosed by detecting of cataplexy. In the pre puberty cases that were suspected of having cataplexy, we asked the physicians to send movie records of their attacks to our laboratory and to measure CSF orexin for diagnosis of narcolepsy-cataplexy, accompanying with the standard PSG and MSLT studies for all age group of patients.

Idiopathic narcolepsy often occurs in the age of adolescence and young adult [17]. While cases of pre puberty children are rare, cases with symptomatic narcolepsy due to NPC have been sometimes reported [2]. When narcolepsy-cataplexy is accompanied by neurological abnormalities such as dystonia and VSSP, a diagnosis of NPC should be considered according to current diagnosis algorithm. Our results of orexin level measurements indicate that orexin can be an early alert of potential NPC. If patients who showed low or intermediate orexin level from initial clinical suspicion, confirming of cataplexy, they should undergo more tests such as gene and biomarker tests during the initial screening. Furthermore, quantification of orexin might be helpful for therapy monitoring after the patients started a treatment. Cataplexy was reported in 26% of all NPC patients and was more often recorded among late-infantile onset (50%) and juvenile onset patients (38%) [6]. In the cases with early-infantile and pro/perinatal onset, cataplexy was not outstanding, because these children had not started standing or walking.

Orexins are hypothalamic neuropeptides involved in various fundamental hypothalamic functions including sleep/wake control, energy homeostasis, and autonomic and neuroendocrine functions [18–20]. Orexin containing neurons are located exclusively in the lateral hypothalamic area (LHA). Since orexin deficiency in narcolepsy is also tightly associated with human leukocyte antigen (HLA) DR2/DQ6 (DQB1*0602) positivity, an acquired cell loss of orexin-containing neurons with autoimmune process is suggested in “idiopathic” cases of narcolepsy [15, 21, 22]. “Idiopathic narcolepsy” is defined as narcolepsy cases unassociated with apparent radiographical or clinical evidence of brain pathology apart from sleep-related abnormalities. Orexin deficiency in the brain can be determined clinically via CSF measures with orexin-A levels in healthy subjects above 200 pg/ml regardless of gender, age (from neonatal to 70s), and time of the CSF collections [9, 14, 15, 23]. Due to the specificity and sensitivity of low CSF orexin levels (less than 110 pg/ml or 30% of the mean normal levels), narcolepsy–cataplexy is high among various sleep disorders [22, 24], and CSF orexin measures were a diagnostic criteria for narcolepsy–cataplexy in the 2nd and narcolepsy type1 in 3rd editions of the International Classification of Sleep Disorders (ICSD-3) [7].

EDS and cataplexy were main symptoms of idiopathic narcolepsy-cataplexy (=NT1, 10,12,14). NPC was known as one of the causes of symptomatic narcolepsy. While cataplexy was often found, EDS was rarely found in this disease of pre-puberty children [2, 25]. Although cataplexy was usually found in idiopathic narcolepsy cases with low orexin levels, intermediate orexin levels were also seen in the cases of NPC with cataplexy, as reported in this study. In our current study, the low or intermediate orexin levels were seen in the patients of NPC with cataplexy.

Cataplexy is caused by neuronal dysfunction of orexin system [26], however, it is not well elucidated why pathophysiology of NPC induces this neuronal dysfunction. Noradrenergic system from orexin neuron was found to be more important for the control of cataplexy by pharmacological examinations [27]; however, recently serotonergic system from orexin neuron is thought to be more involved in the control of cataplexy by bio-molecular examinations [26]. However, it was not known why the intermediate orexin levels in the patients with NPC cause cataplexy. The pathophysiology of NPC would damage orexin and related neurons and decline their functions.

Among symptomatic narcolepsy, Prader-Willi syndrome, myotonic dystrophy type1 and NPC were known as congenital and developmental disorders [25]. We reported that orexin levels with Prader-Willi syndrome and myotonic dystrophy type1 were higher than idiopathic narcolepsy and lower than idiopathic hypersomnia [28, 29]. The orexin levels of NPC were also same as above two diseases (Fig. 2).

We experienced an 8 years old Japanese boy who had drop attacks (Case3). We suspected him to have cataplexy and thus obtained measurement of intermediate orexin level. We also performed DNA analysis and found a well-known NPC1 gene mutation that causes a unique phenotype of NPC, which has been limited to a single Acadian ancestor in Nova Scotia, Canada in 2006 [30].

The ease of access to genetic testing as the first line investigation for neurometabolic disorders has been increasing; however, the orexin measurements could be a good candidate as a biomarker for accurate diagnosis, clinical evaluation with proper treatments, and approach to pathophysiology of narcolepsy-cataplexy and cholesterol metabolism.

### Treatment of NPC

NPC had no effective treatment [31], but we started to use miglustat from 2009 in EU and 2012 in Japan [32]. Miglustat inhibits glucosylceramide synthase, an essential enzyme for the synthesis of most glycosphingolipids (it forms glucosylceramide and accumulates within the macrophages). In this study, former 1–5 cases were untreated by miglustat, and latter 6–10 cases were treated. In two cases with cataplexy (case 3, 4) without miglustat treatment, orexin levels at the onset were intermediate, which became lower in the later period (Fig. 1). Case 6 reduced cataplexy and increased orexin level. Three other cases without cataplexy with miglustat treatment remained as normal orexin levels. It is not clear how miglustat works in favor of cataplectic patients as well as increasing the orexin level. There is a possibility of different sensitivities for miglustat treatment depending on each patient.

### Differentiation of epilepsy

On the other hand, cataplexy is often misdiagnosed as epilepsy, especially as absence seizures. Confusingly, some anti-epileptic medications, such as carbamazepine and clonazepam would reduce cataplexy at moderate or higher dosage [27]. In such cases, accurate diagnoses would be delayed. Tricyclic antidepressant (TCA) and Serotonin & Norepinephrine Reuptake Inhibitors (SNRI) would be more useful for cataplexy even in lower dosage [27]. For cases that are misdiagnosed as epilepsy, these anti-epileptic medications may reduce the attacks. However, they may not improve the orexin level, which could further exacerbate the pathophysiology. Therefore it is crucial that the patients are properly diagnosed in order to ameliorate the conditions from the early phase.

### Limitation

The data of some cases were obtained several years before NPC neurological severity scores were introduced. Although slightly different, their neurological severity is available in forms of orexin level measurements. Also, detailed data of biochemical and neurological examinations during pre and post diagnosis are lacking in some cases.

## Conclusion

In pre-puberty children cases that were suspected of cataplexy by movie records, we recommended examination of CSF orexin and NPC. These NPC patients showed cataplexy without EDS and they had normal results of EEG sleep recording as usual. In the cases with low or intermediate orexin levels, miglustat treatment proved to be effective, stabilizing and/or improving the general condition and increasing some of the patients’ orexin levels. Cases without the treatment showed decrease in orexin level, as well as worsened conditions. It has not been elucidated why intermediate or low orexin levels in the patients with NPC caused cataplexy; however it is clear that the pathophysiology of NPC would damage orexin and related neurons, and decline their functions. If the symptom of cataplexy is observed during initial clinical suspicion, and orexin measurements should be included confirming of this symptom, as well as suggesting for more specific tests. While orexin measurements cannot provide prompt diagnosis, it can serve as an early alert for potential NPC. Our study suggests that orexin measurements could be a marker of therapy monitoring during the treatment.

## Data Availability

Data sharing is applicable to this article as datasets were generated or analysed during the current study.
